# Trading quality for quantity? Evidence from patient level data in China

**DOI:** 10.1371/journal.pone.0257127

**Published:** 2021-09-16

**Authors:** Jinglin Song, Chen Chen, Shaoyang Zhao, Leming Zhou, Hong Chen

**Affiliations:** 1 Department of Public Economic System and Policy, School of Public Administration, Southwestern University of Finance and Economics, Chengdu, Sichuan, China; 2 Department of Finance, School of Public Finance & Economics, Shanxi University of Finance and Economics, Taiyuan, Shanxi, China; 3 Department of Economics, Sichuan University, Chengdu, China; 4 Computer Science and Information Technology College of Chongqing Post and Telecommunication, Chongqing, China; 5 Department of Statistics and Development Research, Chongqing Health Information Center, Chongqing, China; 6 Chongqing Institute of Translational Medicine, University of Chinese Academy of Sciences, Chongqing, China; Ball State University, UNITED STATES

## Abstract

In China, overcrowding at hospitals increases the workload of medical staff, which may negatively impact the quality of medical services. This study empirically examined the impact of hospital admissions on the quality of healthcare services in Chinese hospitals. Specifically, we estimated the impact of the number of hospital admissions per day on a patient’s length of stay (LOS) and hospital mortality rate using both ordinary least squares (OLS) and instrumental variable (IV) methods. To deal with potential endogeneity problems and accurately identify the impact of medical staff configuration on medical quality, the daily air quality index was selected as the IV. Furthermore, we examined the differential effects of hospital admissions on the quality of care across different hospital tiers. We used the data from a random sample of 10% of inpatients from a city in China, covering the period from January 2014 to June 2019. Our final regression analysis included a sample of 167 disease types (as per the ICD-10 classification list) and 862,722 patient cases from 517 hospitals. According to our results, the LOS decreased and hospital mortality rate increased with an increasing number of admissions. Using the IV method, for every additional hospital admission, there was a 6.22% (*p* < 0.01) decrease in LOS and a 1.86% (*p* < 0.01) increase in hospital mortality. The impact of healthcare staffing levels on the quality of care varied between different hospital tiers. The quality of care in secondary hospitals was most affected by the number of admissions, with the average decrease of 18.60% (*p* < 0.05) in LOS and the increase of 6.05% (*p* < 0.01) in hospital mortality for every additional hospital admission in our sample. The findings suggested that the supply of medical services in China should be increased and a hierarchical diagnosis and treatment system should be actively promoted.

## Introduction

In the past two decades, China has continued to push forward its healthcare reforms through rapid growth in health expenditure and medical insurance coverage. By the end of 2018, the total annual expenditure on health in China was close to six trillion yuan, accounting for approximately 6.6% of its gross domestic product, as reported by the *China Health Statistical Yearbook* for 2019. Meanwhile, More than 1.3 billion people were covered by basic medical insurance in China according to the *Blue Book of Health Care—Annual Report on China’s Health Care Development* of 2020, a coverage rate of more than 97%. The increasing demand for medical services combined with the slow growth of medical staff numbers has led to an increasing burden on Chinese medical staff. Overwork not only damages their physical and mental health, but may also negatively impact the quality of medical services. In recent years, research on the relationship between healthcare staffing level and quality of care has focused mainly on some European and American countries, with little research in developing countries. Most scholars have perceived a significant positive correlation between healthcare staffing level and quality of care. This means that a patient experiences better outcomes when there are more medical staff members and care time [1–10]. Therefore, it is particularly important to study the impact of the continuous growth in demand for medical services or that of limited medical resources on the quality of medical services [11, 12].

**Fig 1:**
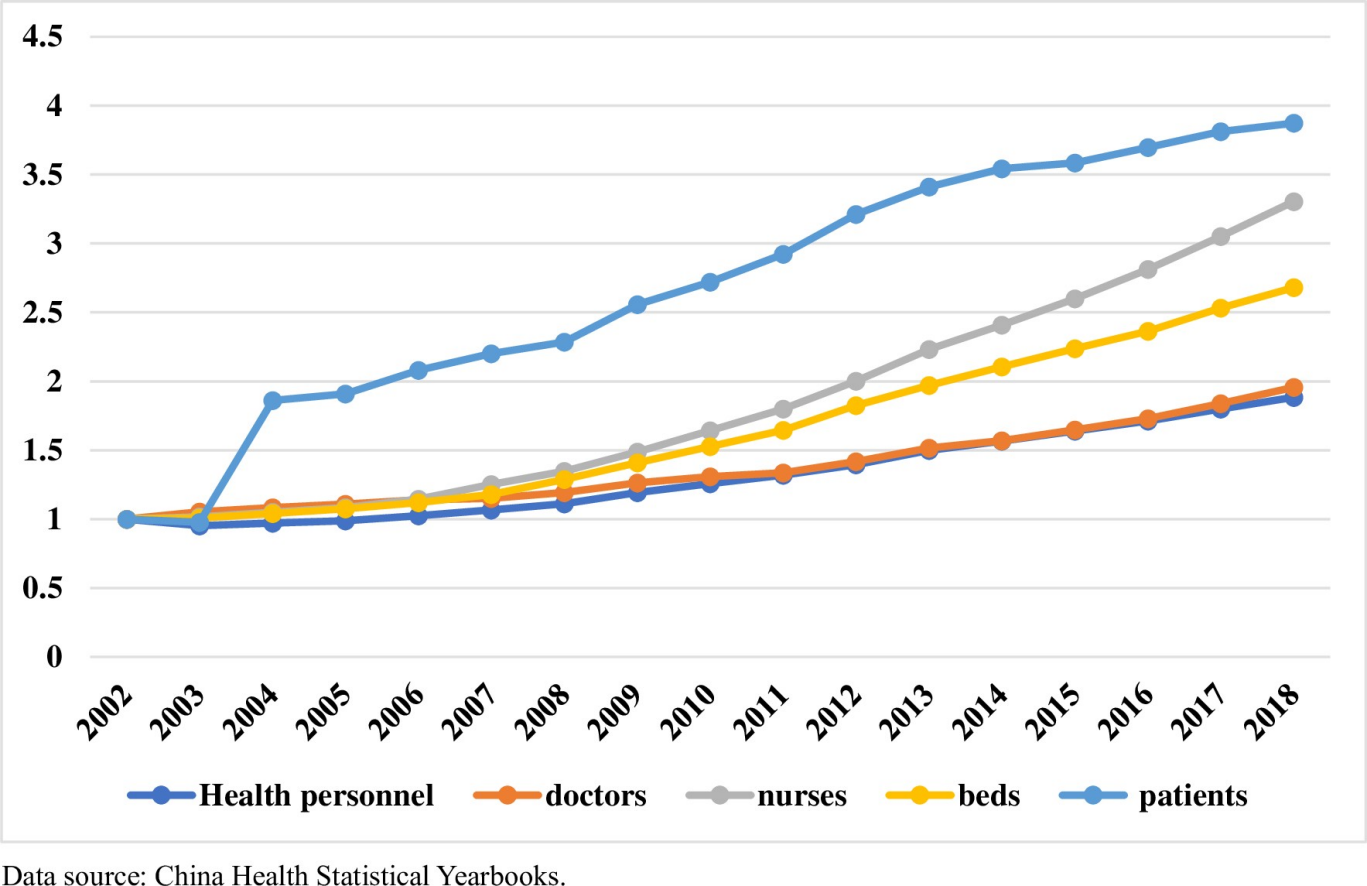
Health personnel, beds, and patient statistics in China investigated from 2002 to 2018. Y-axis represents annual growth rates.

**Fig 2:**
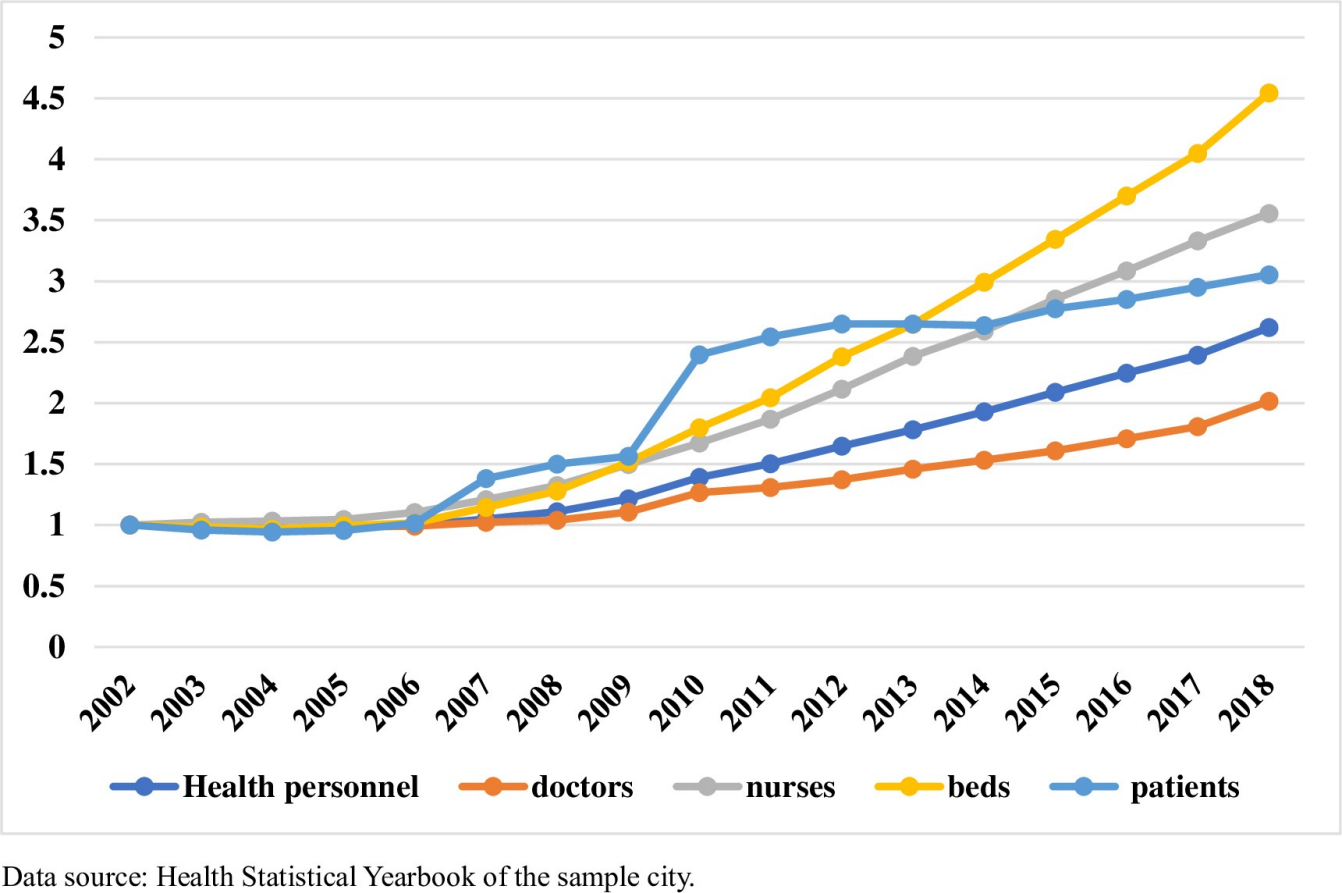
Health personnel, beds, and patient statistics in city investigated from 2002 to 2018. Y-axis represents annual growth rates.

**Fig 3:**
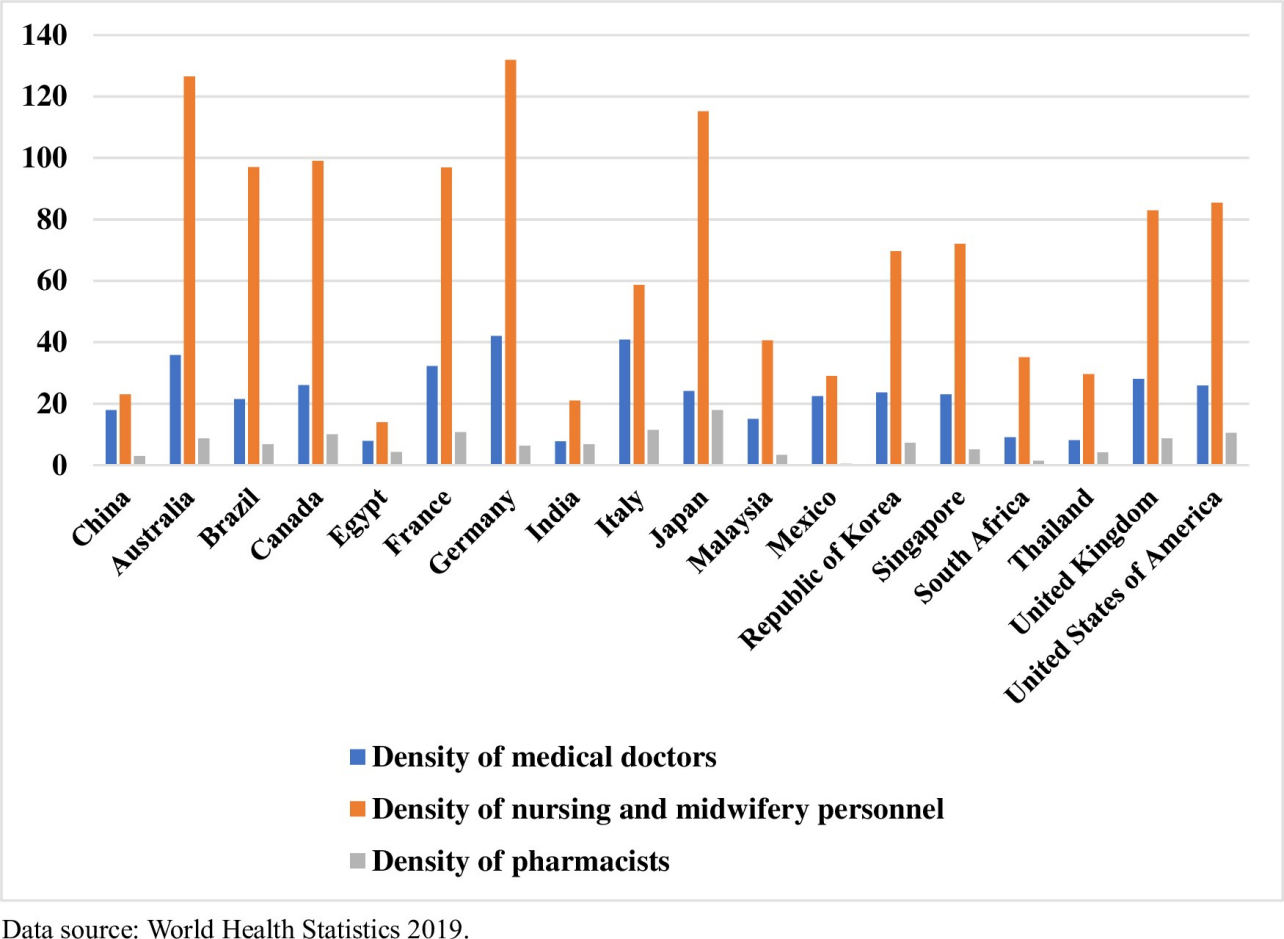
Average density of medical workers (per 10,000 population) for certain countries between 2009 and 2018.

Fig 1 shows that the number of patients receiving care at medical institutions in China increased from 2.1 billion in 2002 to 8.3 billion in 2018. The number of patients increased by about three times in just 16 years. Meanwhile, compared to 2002, the number of beds increased by about 1.7 times in 2018, but the number of health practitioners nationwide increased by only 90%, and the number of doctors also increased by only about 90% in 2018. Fig 2 highlights the situation in the city this study investigated, where the growth in the number of healthcare workers has been much slower than the growth in the number of patients. Fig 3 shows the average density of medical workers (number of medical workers per 10,000 population) of each country for the years 2009 to 2018, which shows that the world’s more developed countries exhibit higher densities of physicians, nurses, and pharmacists than those in China. At present, the distribution of medical resources in China is relatively unequal. resource Most quality medical resources have been concentrated in large general hospitals, and patients can freely self-refer to upper-tier hospitals according to patients’ requirements; as a result, medical and nursing staff working in large general hospitals are constantly overloaded [13]. In terms of working hours, the white paper on the practice status of Chinese physicians in 2014 showed that nearly half of the doctors in the country worked more than 40 hours per week. A total of 92% of doctors in tertiary hospitals needed to work overtime, and 72% of such doctors worked on average more than 60 hours every week [14, 15]. In terms of workload, in 69% of province-level hospitals, an average doctor sees 50 or more patients each day, and 46% of specialists see 100 or more patients each day [16]. Meanwhile, the number of patients cared for by night nurses was approximately three times the number of daytime patients [17]. A large body of literature has found that higher mortality rates can result from increased loads or inadequate healthcare staffing levels [18, 19]. In comparison to doctors abroad, doctors in China take on not only daily heavy clinical work, but also many teaching and research tasks [20]. In the context of high workloads and the relative inadequacy of healthcare staffing levels and healthcare resources, it is common to observe shortened lengths of stay in hospitals (patient hospital days) and early discharges of patients [21, 22]. Some studies have showed that longer hospital stays could indicate worse quality of care [23], and some studies also showed that there was no direct correlation between the length of stay and the quality of medical services. In addition, these phenomena also lead to an increased risk of in-hospital mortality and post-discharge mortality [24–28], which affect people’s health and the efficiency of medical resources use. We explored the changes in the quality of healthcare services brought about by the increased workload of healthcare workers due to the increase in the number of admissions. Because of the increased workload of healthcare workers, along with the tight supply of medical resources such as hospital beds, healthcare workers may discharge patients early before they fully meet discharge criteria. Therefore, we believe that a reduction in the number of hospitalization days would negatively impact the quality of medical services.

In China, patients’ medical decisions are largely voluntary, and coupled with the inadequacy of the country’s primary care system, patients are free to visit hospitals at all levels without appointments or referrals from primary care physicians, and hospital personnel have limited or little control over the number of patients they serve in a day. As a result, the contradiction between the people’s high demand for medical services and the inadequate supply of medical resources is becoming increasingly serious, making it more difficult to access medical care on the one hand, and leading to an increasing burden on China’s medical and nursing staff on the other. Overload not only damages the physical and mental health of healthcare workers, but may also adversely affect the quality of healthcare. This study investigated whether the strain on healthcare resources affects the quality of health care services, an important issue that has not been adequately addressed in the relevant domestic literature [11, 12]. We used data from a 10% random sample of inpatients and annual reports of health care providers in a city in China from January 2014 to June 2019 and developed ordinary least squares (OLS) and instrumental variable (IV) econometric models to analyze the impact of physician human resources allocation on the quality of healthcare services.

The contributions of this study to the existing body of research are reflected in the following aspects: First, the relevant literature has discussed more on the problem of expensive access to healthcare, and very few have discussed the difficulties in accessing health care, especially the consequences of such difficulties in accessing healthcare. Against the backdrop of increasing demand for healthcare, the slow growth of human resources for health, and the corresponding quality of healthcare services, such as decreasing hospitalization days and increasing mortality rates, can be detrimental to people’s health. We used detailed microdata to analyze the impact of the allocation of human capital of health care workers on the quality of health care services; second, this study employed instrumental variables to deal with estimation bias that arises from endogenous changes in the shortage of health care resources. Since the main target population of this study is patients who were not directly affected by air pollution, we chose the daily air quality index (AQI) as the instrumental variable for the number of admissions to address the endogenous bias in the results and to make the estimates more reliable. Third, we compared the difference in the impact of medical staff allocation on the quality of medical services among different hospital tiers. It was found that the medical service quality of secondary hospitals is most affected by the number of admissions, that is, the balance of medical and health resources in hospitals at all levels would affect the quality of medical and health services.

## The hypothesis

### The number of admissions and the quality of medical services

Many studies have found that the more health care workers and the longer the care provided, the better the corresponding treatment outcomes [3, 5–8, 10]. In this study, we assumed that the number of health care workers were not prone to significant change in a short period of time. With larger number of patients corresponded to larger number of health care worker to treat and care for, the worse treatment outcomes. Our explanatory variable of interest—the number of hospital admissions per day—represented the individual’s health seeking behaviors, was complex and could be influenced by many factors, such as educational and economic level, health belief, peer influence and so on [29, 30]. And some of these factors might also affect the quality of health care services. Nevertheless, we lacked reliable estimates of the effect of hospital admissions on quality of health care services. Therefore, using the number of hospital admissions per day as an explanatory variable in the regression may lead to a certain degree of bias in the results.

### The number of admissions and the air quality index

Short term exposure to air pollutants was associated with the majority of risk of hospital admission except for injury and poisoning [31]. Therefore, we hypothesized that the daily air quality index (AQI) would affect patients’ health seeking behavior, which leads to change in hospital admissions. Meanwhile, there was a strong relationship between AQI and the number of hospital admissions per day (S2 File). Differences in air pollutant index were not the only, or even the main cause of variation in the number of hospital admissions. For our empirical approach, all we need is that they were a source of exogenous variation.

### The air quality index and the quality of medical services

The exclusion restriction implied by our instrumental variable regression was that the AQI had no effect on length of stay and hospital mortality. Based on available literatures, air pollution had direct correlations with length of stay and mortality from the patient who had respiratory diseases or heart diseases [32–37]. And the World Health Organization (WHO) has stated that outdoor air pollution-related premature deaths were due to ischaemic heart disease, strokes, chronic obstructive pulmonary disease and acute lower respiratory infections and lung cancer [38]. As far as we know, there was no direct evidence showed that AQI had direct correlations with length of stay and hospital mortality from other disease types. Therefore, in our study, we assumed that the length of stay and hospital mortality for patients without stroke, heart disease, lung cancer and respiratory diseases were not affected by AQI. For example, one patient suffers from uterine smooth muscle tumor and the severity of his/her disease is not affected by air quality, which means AQI does not directly affect the quality of health care services for this patient.

Therefore, we excluded the patients whose main diagnosis were the above four kind of disease types in our empirical study.

## Data and methods

### Data

The data used in this study were from 10% random samples on inpatient medical records cover sheet in a city of China from January 2014 to June 2019. The city is located in the inland southwest of China and the upper reaches of the Yangtze River; it has a permanent resident population of 31.24 million. The data included the type of insurance, disease diagnosis information, gender, age, length of stay, cost of hospitalization, and way of leaving the hospital. Because the quality of medical services is affected not only by the personal characteristics of patients but also by medical institutions, we used the statistical information of the annual report of the city’s medical and health service institutions, and combined the institutional data and insurance data. Medical institution information includes hospital tier, ownership, and scale of various assets, among others. It should be noted that the city selected for this study is a representative sample. By comparing the national macro database with the current micro database, we found that there was no significant difference between the data of this city and those from other databases in terms of personal characteristics, lengths of stay, and disease mortality, which increases the generalizability of this study’s conclusions. In addition, the air quality of the city we investigated is better than that of most other parts of China. Therefore, our results were underestimated.

These inpatient data were used to explore only the impact of hospital overcrowding on quality of care. All the personal information was de-identified and only the objective variables could be analyzed under restricted governance of policies. All the data used in this study were taken from the China Regional Health Information Platform, which collects administrative type of medical data. We have signed a confidentiality agreement with the China Regional Health Information Center. All research procedures were approved by the Regional National Health Committee of China.

Air quality index (AQI) is a numerical scale used for demonstrating air quality, which could provide indicator for the correlation between air pollution exposure and risk for health. We extracted the daily 24-hour mean AQI over the study period from the air quality data platform operated by China National Environmental Monitoring Center (https://www.aqistudy.cn/historydata/). Furthermore, in the robustness checks, to adjust potential correlation of weather conditions, daily 24-hour mean temperature, humidity, wind speed, atmospheric pressure, precipitation and sunshine hours were obtained from the China Meteorological Data Service Center (http://data.cma.cn/en).

Because the number of admissions and health status of patients with stroke, heart disease, lung cancer, and respiratory diseases are directly affected by air pollution [28, 39, 40], we identified the disease types of patients according to the top four of the disease codes (as per the ICD-10 classification list), deleted the four kinds of diseases that are greatly affected by air pollution, and selected the disease types with a sample size greater than 100, a total of 167 types (S1 Table). The final sample size was composed of 862,722 patients, including 517 hospitals. It should be noted that given the degree of exclusivity of resources such as examination equipment and beds within entire hospitals, patients with stroke, heart disease, lung cancer, and respiratory disease were included in the calculation of our key explanatory variable hospital admissions, but records of consultations for these four types of diseases were removed from the sample data.

### Methods

We used Stata 15 software to investigate the impact of changes in human resources allocation of doctors caused by different types of admissions on the quality of medical services, using data from patients and hospitals. The specific empirical model was as follows:

$$Y_{ijt}=\beta_{0}+\beta_{2}N_{tj}+\gamma X_{i}+\lambda_{t}+\mu_{j}+\varepsilon_{itj}$$ (1)

where *Y_ijt_* represents the quality of the medical services of patient *i* attending hospital *j* at time *t*. Two indicators, namely, length of stay and in-hospital death, were used to measure the quality of medical service. In the regression analysis, we used the log-change method to transform the length of stay (LOS) variable. This method involves taking the logarithm of the explanatory variable and then placing the logarithmic values into a multivariate linear regression model. The hospital mortality rates were measured by identifying patients that died based on the “mode of discharge” variable, and then calculating the number of deaths as a percentage of total discharges. *N_tj_* is the key explanatory variable, which is the number of patients admitted per day calculated based on each hospital according to the natural changes of patients’ admission time, to measure the load intensity of medical staff. *X_i_* is the patient’s characteristics that affect the patient’ s health outcomes, including age, gender, marital status, employment status, admission route, patient had been hospitalized before, health insurance status of medical expenses, and disease diagnosis type. *λ_t_* is the time fixed effect (year, month, and holiday), *μ_j_* represents the hospital fixed effect, and *ε_itj_* is a random error term. Considering that the load intensity of medical staff is related only to the hospital, the number of patients was calculated according to each hospital. In addition, there is a relationship between the number of patients and holidays [28]. The holidays in this study included Saturdays, Sundays, and various national statutory holidays (e.g., Spring Festival and Mid-Autumn Festival).

In a statistical model, a variable is said to be endogenous when there is a correlation between the variable and the error term. Hospital admissions, the main explanatory variable, are determined by various factors that may influence the explained variable—the quality of medical services. Therefore, to deal with such possible endogenous problems and accurately identify the impact of medical staff configuration on medical quality, the daily air quality index (AQI) was selected as the IV, and a two-stage least squares regression was performed. The first-stage regression equation was as follows:

$$N_{tj}=\beta_{0}+\beta_{2}AQI+\gamma X_{i}+\lambda_{t}+\mu_{j}+\varepsilon_{itj}$$ (2)

Here, AQI measures the degree of daily pollution. The greater the AQI, the higher air pollution, and the worse the air quality. The potential hypothesis in Eq (2) is that AQI affects the number of hospital admissions at the time and medical institution levels. Because the patients’ health seeking behavior is the result of self-selection in China, namely they could freely to choose any tiers of medical institutions. If people feel sick on the high AQI, they could self-refer to higher-level of providers, which increases the average daily number of hospital admission, especially for higher-level hospitals. In addition, from the selection of instrumental variables the environmental pollution index is highly correlated with the number of visits for stroke, heart disease, lung cancer and respiratory diseases; second, the environmental pollution index is an exogenous variable that is not correlated with other explanatory variables in the model; and third, the health status of patients with stroke, heart disease, lung cancer and respiratory diseases is highly influenced by air pollution. We excluded the quality of medical services data for these four diseases, so the air pollution index and the quality of medical services for patients in this sample were not correlated. In summary, our selection of AQI as an instrumental variable for the number of visits satisfied the conditions for the selection of instrumental variables.

### Results

Table 1 shows the patient information variables and related variables of medical institutions. For the whole sample, 55.55% were male patients, 88% were over 18 years old, 26.08% had urban employees’ basic medical insurance, 27% had urban residents’ basic medical insurance, and 12% had new rural cooperative medical insurance. Among the sample, 211,127 (24.5%) were admitted to the emergency department, and 509,095 (59.0%) were admitted to general hospitals. The most prevalent employment status was farmers (23.42%), and 660,632 (76.6%) people were married. Most patients (87.52%) attended a tertiary or secondary hospital, and only 2.6% chose a primary medical institution.

**Table 1:** Basic characteristics of patients.

	N	2014	2015	2016	2017	2018	2019
**N**	862,722	94,346 (10.94)	111,435 (12.92)	164,510 (19.07)	189,983 (22.02)	196,037 (22.72)	106,41 (12.33)
**Gender**							
Male	479,181	50,523 (53.55)	59,875 (53.73)	92,627 (56.34)	107,823 (56.77)	109,339 (55.78)	58,994 (55.44)
Female	383,541	43,822 (46.45)	51,558 (46.27)	71,794 (43.66)	82,100 (43.23)	86,680 (44.22)	47,410 (44.56)
**Age group (years)**							
≤18	100,953	13,086 (13.87)	14,688 (13.18)	18,994 (11.55)	21,198 (11.16)	22,039 (11.24)	10,948 (10.29)
19–59	456,093	49,284 (52.24)	58,056 (52.10)	88,311 (53.68)	101,311 (53.33)	103,009 (52.55)	56,122 (52.74)
≥60	305,676	31,976 (33.89)	38,691 (34.72)	57,205 (34.77)	67,474 (35.52)	70,989 (36.21)	39,341 (36.97)
**Health insurance status**							
UEBMI	225,037	26,659 (28.26)	31,149 (27.95)	44,685 (27.16)	48,431 (25.49)	48,637 (24.81)	25,476 (23.94)
URBMI	228,619	21,316 (22.59)	25,938 (23.28)	38,795 (23.58)	48,461 (25.51)	59,595 (30.40)	34,514 (32.43)
NRCMS	103,904	13,337 (14.14)	13,561 (12.17)	20,739 (12.61)	24,368 (12.83)	20,868 (10.64)	11,031 (10.37)
Others	305,162	33,034 (35.01)	40,787 (36.60)	60,291 (36.65)	68,723 (36.17)	66,937 (34.15)	35,390 (33.26)
**Route of hospital admission**							
Admitted through Emergency	211,127	34,322 (36.38)	50,970 (45.74)	100,416(61.04)	116,486(61.31)	132,564(67.62)	74,337 (69.86)
Admitted through Outpatient	509,095	40,208 (42.62)	35,059 (31.46)	35,261 (21.43)	39,253 (20.66)	40,848 (20.84)	20,498 (19.26)
Other	142,500	19,816 (21.00)	25,406 (22.80)	28,833 (17.53)	34,244 (18.02)	22,625 (11.54)	11,576 (10.88)
**Employment status**							
Unemployed	54,841	4,960 (5.26)	5,667 (5.09)	10,215 (6.21)	12,866 (6.77)	13,423 (6.85)	7,710 (7.25)
Farmer	202,069	23,570 (24.98)	26,633 (23.90)	35,883 (21.81)	41,856 (22.03)	46,990 (23.97)	27,137 (25.50)
Retired	57,489	7,580 (8.03)	8,198 (7.36)	10,582 (6.43)	11,484 (6.04)	12,462 (6.36)	7,183 (6.75)
Self-employed	28,928	2,469 (2.62)	3,336 (2.99)	5,541 (3.37)	6,413 (3.38)	7,169 (3.66)	4,000 (3.76)
Employed	108,304	11,907 (12.62)	12,217 (10.96)	19,976 (12.14)	24,515 (12.90)	25,743 (13.13)	13,946 (13.11)
Other	411,091	43,860 (46.49)	55,384 (49.70)	82,313 (50.04)	92,849 (48.87)	90,250 (46.04)	46,435 (43.64)
**Marriage status**							
Never married	144,080	17,782 (18.85)	20,222 (18.15)	28,036 (17.04)	31,379 (16.52)	31,018 (15.82)	15,643 (14.70)
Married	660,632	72,405 (76.74)	85,899 (77.08)	125,332(76.19)	143,637(75.61)	150,453(76.75)	82,906 (77.91)
Widowed	19,140	1,392 (1.48)	1,865 (1.67)	3,178 (1.93)	4,420 (2.33)	5,188 (2.65)	3,097 (2.91)
Divorced	9,408	825 (0.87)	1,106 (0.99)	1,535 (0.93)	1,926 (1.01)	2,526 (1.29)	1,490 (1.40)
Other	29,462	1,942 (2.06)	2,343 (2.10)	6,429 (3.91)	8,621 (4.54)	6,852 (3.50)	3,275 (3.08)
**Patient had been hospitalized before**							
No	751,620	90,406 (95.82)	104,912(94.15)	149,971(91.16)	163,134(85.87)	159,984(81.61)	83,213 (78.20)
Yes	111,102	3,940 (4.18)	6,523 (5.85)	14,539 (8.84)	26,849 (14.13)	36,053 (18.39)	23,198 (21.80)
**Length of stay (days)**							
<5	303,215	30,709 (32.55)	37,288 (33.46)	57,995 (35.25)	68,627 (36.12)	70,153 (35.79)	38,443 (36.13)
5–10	366,099	39,918 (42.31)	46,877 (42.07)	70,285 (42.72)	81,026 (42.65)	83,624 (42.66)	44,369 (41.70)
11–15	93,809	11,828 (12.54)	13,194 (11.84)	17,888 (10.87)	19,908 (10.48)	20,278 (10.34)	10,713 (10.07)
>15	99,599	11,891 (12.60)	14,076 (12.63)	18,342 (11.15)	20,422 (10.75)	21,982 (11.21)	12,886 (12.11)
**Hospitalization level**							
Primary	22,457	6 (0.01)	63 (0.06)	5,439 (3.31)	6,340 (3.34)	6,821 (3.48)	3,788 (3.56)
Secondary	385,876	45,207 (47.92)	54,053 (48.51)	74,878 (45.52)	82,858 (43.62)	83,574 (42.63)	45,306 (42.58)
Tertiary	369,150	49,094 (52.04)	56,914 (51.07)	66,342 (40.33)	75,324 (39.65)	79,050 (40.32)	42,426 (39.87)
Others	85,200	39 (0.04)	405 (0.36)	17,840 (10.85)	25,437 (13.39)	26,588 (13.56)	14,891 (13.99)

*Notes*: N: number of patients; NRCMS: the new rural cooperative medical insurance; UEBMI: the basic medical insurance for urban employees; URBMI: the basic medical insurance for urban residents.

Table 2 presents the descriptive statistics of the main explanatory variables. The number of patients in the city has been increasing every year since 2014. The average air pollution index of the sample was 70.51, and the average length of stay and death rate of patients were 9.52 days and 0.04, respectively.

**Table 2:** Descriptive statistics of air quality index, length of stay, hospital mortality rates and number of patients from January 2014 to June 2019.

	AQI	Length of stay	Hospital Mortality rates	Number of patients
**2014**	86.83 (45.98)	9.07 (11.97)	0.11 (0.31)	94,346
**2015**	77.93 (40.99)	9.2 (13.80)	0.09 (0.28)	111,435
**2016**	74.02 (31.47)	9.27 (21.25)	0.04 (0.19)	164,510
**2017**	67.51 (34.73)	9.28 (18.79)	0.03 (0.18)	189,983
**2018**	62.8 (28.28)	9.59 (19.79)	0.01 (0.12)	196,037
**2019**	62.42 (27.23)	10.9 (25.87)	0.01 (0.09)	106,411

*Notes*: Standard errors in parentheses; AQI: air quality index.

Table 3 shows the regression results for the log length of stay. The first column of the OLS regression controlled only the number of hospital admissions and the fixed effect of time. We observed that the number of inpatients had a significant positive effect on the log length of stay. When the control variables of patients and hospitals and the fixed effect of hospitals were added gradually, the coefficient of the number of admissions gradually decreased, but was still significantly positive (second and third columns). Meanwhile, the empirical results of the control variables introduced a significant impact on the log length of stay, and with the introduction of patient and hospital characteristic variables, the goodness of fit of the model gradually increased.

**Table 3:** Regression results of log length of stay.

	OLS			IV		
	(1)	(2)	(3)	(4)	(5)	(6)
**N**	0.0028 (0.0001)***	0.0027 (0.0001)***	0.0018 (0.0001)***	-0.0303 (0.0117)**	-0.0484 (0.0118)***	-0.0622 (0.0130)***
**Patient X**						
Age		0.0043 (0.0001)***	0.0044 (0.0001)***		0.0036 (0.0002)***	0.0044 (0.0001)***
Gender(ref. female)		0.0323 (0.0016)***	0.0278 (0.0016)***		0.0484 (0.0041)***	0.0306 (0.0019)***
Hospital admission		-0.0128 (0.0009)***	0.0011 (0.0012)*		0.0287 (0.0094)***	0.0016 (0.0014)
**Payment (ref. URBMI)**						
UEBMI		-0.0600 (0.0021)***	-0.0585 (0.0022)***		0.0613 (0.0026)***	0.0595 (0.0025)***
NRCMS		0.0027 (0.0024)	-0.0154 (0.0029)***		-0.0128 (0.0040)***	-0.0264 (0.0039)***
Others		-0.0120 (0.0019)***	-0.0040 (0.0024)*		-0.1328 (0.0278)***	-0.0124 (0.0032)**
**Employment status (ref. employed)**						
Unemployment		-0.0391 (0.0033)***	-0.0132 (0.0033)***		-0.0813 (0.0104)***	-0.0073 (0.0040)*
Farmer		-0.0412 (0.0028)***	-0.0569 (0.0029)***		-0.0642 (0.0064)***	-0.0477 (0.0038)***
Retire		0.0528 (0.0037)***	0.0672 (0.0037)***		0.0952 (0.0107)***	0.0701 (0.0042)***
Self-employment		-0.0037 (0.0042)	-0.0175 (0.0042)***		-0.0143 (0.0055)***	-0.0216 (0.0047)***
Others		-0.0227 (0.0024)***	-0.0427 (0.0025)***		-0.0053 (0.0071)	-0.0371 (0.0030)***
**Marriage status (ref. married)**						
Single		0.0073 (0.0028)***	0.0010 (0.0029)		0.0191 (0.0045)***	0.0158 (0.0046)***
Widowed		-0.0127 (0.0053)**	-0.0140 (0.0051)***		-0.0868 (0.0179)***	-0.0226 (0.0061)***
Divorced		0.1404 (0.0086)***	0.1198 (0.0082)***		0.1165 (0.0140)***	0.1525 (0.0135)***
Others		0.0359*** (0.0042)	-0.0267 (0.0045)***		-0.1717 (0.0478)***	-0.0165 (0.0060)***
**HospitalX (ref. tertiary)**						
Primary			0.3158 (0.1608)**			0.9869 (0.0604)***
Secondary			-0.1276 (0.1248)			0.1516 (0.2016)
Others			-0.2182 (35.4177)			0.4667 (0.1845)**
**First stage**						
AQI	-	-	-	0.0026 (0.0004)***	0.0026 (0.0004)***	0.0021 (0.0002)***
First stage F-test	-	-	-	48.91	54.62	91.46
**Hospitalization times**	No	Yes	Yes	No	Yes	Yes
**ICD10**	No	Yes	Yes	No	Yes	Yes
**Hospital_FE**	No	No	Yes	No	No	Yes
**Year**	Yes	Yes	Yes	Yes	Yes	Yes
**Month**	Yes	Yes	Yes	Yes	Yes	Yes
**Holiday**	Yes	Yes	Yes	Yes	Yes	Yes
**R** ^**2**^	0.1880	0.2063	0.2521	-	-	-
**MSE**	-	-	-	0.6572	0.5661	0.4604
**Obs.**	862,722	862,722	862,722	862,722	862,722	862,722

*Notes*: Standard errors in parentheses.

Significance level:

*** p <0.01

** p <0.05

* p <0.1.

Patient’s personal control variables include age, gender, payment method, admission channel, occupation, marriage, and hospitalization times; Hospital control variables include hospital tier; The results of all the control variables are consistent with expectations; Due to space limitations, no results are reported in the table.

N: number of patients admitted per day; NRCMS: the new rural cooperative medical insurance; UEBMI: the basic medical insurance for urban employees; URBMI: the basic medical insurance for urban residents; AQI: air quality index; MSE: mean square error; Obs.: the number of observations.

Considering the endogeneity problems between the number of hospital admissions and the length of stay, we chose daily AQI as an IV. The last three columns of Table 3 report the estimation results of the IV method and the corresponding first stages. Importantly, a concern with an IV regression is the possible of weak instrument, which based on the strength of first stage equation [41]. The first stages of our IV estimate displayed the strong positive relationship between AQI and the number of hospital admissions (F-test were 48.91, 54.62 and 91.46 in columns (4), (5) and (6), and *p*<0.01), which implied that our first stages have good power and our instrument was not weak. Thus, the daily AQI is an effective IV in an econometric sense. The result of column (4) shows that the estimated coefficient was -3.03%, which was significant at the 1% level after considering the endogeneity of the number of admissions. Columns (5) and (6) shows that adding control variables of patients and hospitals and the fixed effect of hospitals did not change the estimated effect of the number of admissions, and the coefficients were -4.84% and -6.22%, with significant at 1% level. This shows that the log length of stay of patients decreased with an increase in the number of admissions. Compared with the OLS estimation, the coefficient of the IV estimation was negative, which shows that the number of admissions deviated somewhat from the OLS estimation of the log length of stay.

Table 4 presents the regression results for hospital mortality. The first column of the OLS regression controlled only the number of inpatients and the fixed effect of time. We observed that the number of hospitalizations had a significant negative effect on hospital mortality. When the control variables of patients and hospitals and the fixed effects of hospitals were added, the coefficient of the number of hospital admissions was significantly positive (third column), indicating that for each additional admission, the hospital mortality rate of patients increased by approximately 0.0002. The columns (4), (5) and (6) of Table 4 present the estimated results for IV and the corresponding first stages. Similarly, the first stages displayed the strong positive relationship between AQI and the number of hospital admissions (F-test were 49.31, 55.08 and 91.18 in columns (4), (5) and (6), and *p*<0.01). After considering the endogeneity problems of the number of hospital admissions, the corresponding IV estimate coefficients were 0.0146, 0.0151 and 0.0186, which were still significant at the 1% level, indicating that as the number of hospital admissions increased, the increase in patient mortality was robust. Compared with the OLS estimation, the coefficients derived from the IV estimation were higher. In general, the IV results show that the hospital mortality rate of patients was greatly affected by the number of hospital admissions. The higher the number of hospital admissions, the higher the hospital mortality rate of patients.

**Table 4:** Regression results of hospital mortality.

	OLS			IV		
	(1)	(2)	(3)	(4)	(5)	(6)
**N**	-0.0017 (0.0000)***	-0.0016 (0.0000)***	0.0002 (0.0000)***	0.0146 (0.0039)***	0.0151 (0.0037)***	0.0186 (0.0035)***
**Patient X**						
Age		-0.0000 (0.0000)*	-0.0000 (0.0000)		0.0002 (0.0001)***	0.0000 (0.0000)
Gender (ref. female)		0.0032 (0.0005)***	0.0031 (0.0004)***		0.0022 (0.0013)*	0.0024 (0.0005)***
Hospital admission		-0.0565 (0.0004)***	-0.0559 (0.0004)***		-0.0702 (0.0030)***	-0.0568 (0.0004)***
**Payment (ref. URBMI)**						
UEBMI		-0.0089 (0.0007)***	-0.0005 (0.0006)		-0.0078 (0.0009)***	-0.0082 (0.00007)***
NRCMS		-0.0204 (0.0007)***	-0.0020 (0.0006)***		-0.0157 (0.0011)***	-0.0032 (0.0010)***
Others		-0.0405 (0.0006)***	0.0097 (0.0006)***		-0.0082 (0.0087)	-0.0114 (0.0008)***
**Employment status (ref. employed)**						
Unemployment		0.0085 (0.0009)***	0.0180 (0.0008)***		0.0230 (0.0033)***	0.0172 (0.0011)***
Farmer		0.0085 (0.0009)***	0.0108 (0.0007)***		0.0182 (0.0019)***	0.0088 (0.0010)***
Retire		0.0333 (0.0012)***	0.0318 (0.0010)***		0.0201 (0.0033)***	0.0315 (0.0012)***
Self-employment		-0.0047 (0.0010)***	0.0020 (0.0009)**		-0.0028 (0.0015)*	0.0035 (0.0011)**
Others		0.0081 (0.0006)***	0.0144 (0.0006)***		-0.0023 (0.0021)	0.0133 (0.0007)***
**Marriage status (ref. married)**						
Single		0.0022 (0.0008)	0.0005 (0.0007)		0.0065 (0.0014)***	-0.0068 (0.0012)***
Widowed		-0.0089 (0.0013)***	0.0009 (0.0012)		0.0198 (0.0056)***	0.0029 (0.0015)*
Divorced		0.0239 (0.0023)***	0.0183 (0.0019)***		0.0323 (0.0040)***	0.0108 (0.0036)***
Others		0.0345 (0.0015)***	0.0084 (0.0012)***		0.1097 (0.0151)***	0.0086 (0.0017)***
**HospitalX (ref. tertiary)**						
Primary			-0.0161 (0.0053)***			-0.0065 (0.041)
Secondary			0.0144 (D)			-0.0887 (0.0224)***
Others			0.0009 (0.0054)			-0.0100 (0.0096)
**First stage**						
AQI	-	-	-	0.0026 (0.0004)***	0.0026 (0.0004)***	0.0021 (0.0002)***
First stage F-test	-	-	-	49.31	55.08	91.18
**Hospitalization times**	No	Yes	Yes	No	Yes	Yes
**ICD10**	No	Yes	Yes	No	Yes	Yes
**Hospital_FE**	No	No	Yes	No	No	Yes
**Year**	Yes	Yes	Yes	Yes	Yes	Yes
**Month**	Yes	Yes	Yes	Yes	Yes	Yes
**Holiday**	Yes	Yes	Yes	Yes	Yes	Yes
**R** ^**2**^	0.0604	0.1379	0.4203	-	-	-
**MSE**	-	-	-	0.0642	0.0668	0.0385
**Obs.**	862,722	862,722	862,722	862,722	862,722	862,722

*Notes*: Standard errors in parentheses.

Significance level:

*** p <0.01

** p <0.05

* p <0.1.

Patient’s personal control variables include age, gender, payment method, admission channel, occupation, marriage, and hospitalization times; Because there was a degree of collinearity, the standard deviations for the secondary hospital in column (3) are represented by (D); Hospital control variables include hospital tier; The results of all the control variables are consistent with expectations; Due to space limitations, no results are reported in the table.

N: number of patients admitted per day; NRCMS: the new rural cooperative medical insurance; UEBMI: the basic medical insurance for urban employees; URBMI: the basic medical insurance for urban residents; Hospital_FE: hospital fixed effects; AQI: air quality index; MSE: mean square error; Obs.: the number of observations.

Table 5 shows the regression results for the log length of stay for different tiers of hospitals. The OLS results show that regardless of the level of the hospital, the length of stay increased with the number of inpatients, the length of stay increases. Because of the influence of endogeneity, the OLS results had some deviation, thus, the results of IV were revised. In columns (5) and (8), the IV estimated coefficients reported positive impact but statistically insignificant at a conventional level, although the resulting first stages were significant (F-test are 7.09 and 63.31). On the contrary, columns (6) and (7) reported significant negative effect on the log length of stay in secondary and tertiary hospitals, and the corresponding first stages displayed positive relationship (F-test were 11.52 and 62.42, *p*<0.01). Specifically, for each increase in the number of admissions, the length of stay of patients in secondary hospitals decreased by 18.60%, and the results were significant at the 5% level. For patients in tertiary hospitals, the length of stay decreased by 4.83% with each increase in the number of patients, significant at the 1% level.

**Table 5:** Regression results of log length of stay for different hospital tiers.

	(1)	(2)	(3)	(4)	(5)	(6)	(7)	(8)
	Primary hospitals (OLS)	Secondary hospitals (OLS)	Tertiary hospitals (OLS)	Others (OLS)	Primary hospitals (IV)	Secondary hospitals (IV)	Tertiary hospitals (IV)	Others (IV)
**N**	0.0175 (0.0030)***	0.0013 (0.0003)***	0.0017 (0.0002)***	0.0153 (0.0011)***	0.1263 (0.1297)	-0.1860 (0.0765)**	-0.0483 (0.0128)***	0.0160 (0.0254)
**First stage**								
AQI	-	-	-	-	-0.0012 (0.0004)***	0.0007 (0.0002)***	0.0034 (0.0004)***	0.0028 (0.0004)***
First stage F-test	-	-	-	-	7.09	11.43	57.09	63.31
**R** ^**2**^	0.5929	0.1943	0.2448	0.4264	-	-	-	-
**MSE**	-	-	-	-	3.5645	5.3058	26.00	0.9292
**Obs.**	22,457	385,876	369,150	85,239	22,457	385,876	369,150	85,239

*Notes*: Standard errors in parentheses.

Significance level:

*** p <0.01

** p <0.05

* p <0.1.

Patient’s personal control variables include age, gender, payment method, admission channel, occupation, marriage, and hospitalization times; Hospital control variables include hospital tier; The results of all the control variables are consistent with expectations; Due to space limitations, no results are reported in the table.

N: number of patients admitted per day; AQI: air quality index; MSE: mean square error; Obs.: the number of observations.

Table 6 presents the OLS and IV regressions results of hospital mortality for different tiers of hospitals. Columns (2), (6) and columns (3), (7) show that in secondary and tertiary hospitals sample there was a positive correlation between the number of hospital admissions and hospital mortality, even it was insignificant of the OLS coefficient for secondary hospitals. And the OLS estimated coefficients were smaller than the IV estimates, which were similar to the result reported in Table 4. For primary and others medical institutions, the IV estimates reported insignificant correlations between the number of hospital admissions and hospital mortality in columns (5) and (8).

**Table 6:** Regression results of hospital mortality for different hospital tiers.

	(1)	(2)	(3)	(4)	(5)	(6)	(7)	(8)
	Primary hospitals (OLS)	Secondary hospitals (OLS)	Tertiary hospitals (OLS)	Others (OLS)	Primary hospitals (IV)	Secondary hospitals (IV)	Tertiary hospitals (IV)	Others (IV)
**N**	-0.0005 (0.0003)*	0.0001 (0.0001)	0.0001 (0.0000)***	0.0018 (0.0002)***	0.0042 (0.0381)	0.0605 (0.0235)**	0.0138 (0.0030)***	-0.0015 (0.0082)
**First stage**								
AQI	-	-	-	-	-0.0012 (0.0004)***	0.0007 (0.0002)***	0.0034 (0.0004)***	0.0028 (0.0004)***
First stage F-test	-	-	-	-	7.09	11.39	56.87	63.31
**R** ^**2**^	0.5955	0.3556	0.2636	0.6146	-	-	-	-
**MSE**	-	-	-	-	0.0927	0.5856	38.65	0.0521
**Obs.**	22,457	385,876	369,150	85,239	22,457	385,876	369,150	85,239

*Notes*: Standard errors in parentheses.

Significance level:

*** p <0.01

** p <0.05

* p <0.1.

Patient’s personal control variables include age, gender, payment method, admission channel, occupation, marriage, and hospitalization times; Hospital control variables include hospital tier; The results of all the control variables are consistent with expectations; Due to space limitations, no results are reported in the table.

N: number of patients admitted per day; AQI: air quality index; MSE: mean square error; Obs.: the number of observations.

We also explored the robustness of our findings to control for potential correlation of weather conditions with AQI level and the quality of medical services. The IV estimates changed remarkably little when we included controls for the daily 24-hour mean temperature, humidity, wind, pressure, precipitation and sunshine hours and were reported in S3 File. It shows that the results of our instrumental variable were robust.

## Discussion and conclusion

We used the data of 10% randomly sampled inpatients and annual reports of medical and health service institutions in a city of China from January 2014 to June 2019, and we established OLS and IV estimation methods to analyze the impact of human resources allocation of doctors on the quality of medical services. We found that with an increase in the number of admissions, the length of stay decreased and the hospital mortality increased. Comparing the IV estimation with the OLS estimation, we found that neglecting endogeneity leads to underestimation of the impact of medical staff allocation on medical service quality. Previous studies have also found that during long vacations, a decrease in human capital and an increase in workload would lead to an increase in the hospital mortality and incidence rate [42].

At present, China is in a transition period of medical reform, which has resulted in a large medical resources gap. Hospitals cannot rely only on increasing the working hours of medical staff to meet the medical needs of the people, as this has a negative impact on the quality of medical services. Therefore, the managers of the healthcare system, including the government and hospital managers, should pay attention to the quality of medical services, innovate the management system, reform the performance appraisal mechanism, adjust the price system of medical services, improve the overwork status quo of medical staff, improve their job satisfaction and sense of professional achievement, and maintain the quality and safety of medical services. Furthermore, all departments should improve the supervisory mechanism, improve the efficiency of hospital work, and ensure the quality of medical services.

It is worth noting that the distribution of medical resources in hospitals at all tiers is relatively unbalanced. Secondary and tertiary hospitals are the higher tier of hospitals in China, providing higher quality medical services and technical expertise, with advantages in scientific research and equipment. Thus, secondary and tertiary hospitals have more high-quality doctors and other high-quality resources. Meanwhile, they attract more patients seeking visits with a doctor. As a result, the higher the tier of the hospital, the more patients doctors treat every day, and the greater the work intensity of the medical staff [43]. The results of this study show that the quality of medical services in secondary hospitals is most affected by the number of admissions. When the number of admissions increased by one, the average length of stay of patients decreased by 18.60%, and the death rate increased by 6.05%. To improve the imbalance and unfairness of medical resources, all departments in a hospital should actively promote the hierarchical diagnosis and treatment system, strengthen the quality of doctors in primary medical institutions, dredge referral channels, and strive to maximize the use of limited medical resources.

With the development of the world economy, the population aging process has accelerated, and the incidence of various chronic diseases has increased. An increasing number of people visiting hospitals have placed great pressure on hospitals. Although the scale and number of beds in hospitals have been expanding in recent years, the growth rate of medical staff is slow because of understaffing. As a result, hospitals often take more measures to extend the working time of medical staff and reduce the patient’s treatment time to alleviate the gap between the growing demand for medical services and insufficient supply. However, excessive workloads can affect the physical and mental health of medical staff, reduce their enthusiasm for work, and may also increase job burnout, which would negatively impact the quality of medical services and medical safety [44–47].

Our study has several limitations. First, we used random sampling data from a city in China, which may limit the ability to promote relevant recommendations to all developing countries. Second, according to the functions, tasks, facilities, technical construction, medical service quality and comprehensive level of scientific management, hospitals in China are divided into primary, secondary and tertiary hospitals. Based on the analysis of the impact of medical staff allocation on the quality of medical services among different hospital tiers, we put forward relevant suggestions, but for other developing countries, due to the differences in medical systems, the relevant conclusions may not be universal. Finally, because we used administrative data, important variables that affect the quality of medical services, such as the income level of patients, could not be obtained.

Of course, an increase in the number of admissions and a decrease in the number of hospital days are inseparable from the natural population growth rate and the progress of technology; however, according to the *20th in the series of reports on the achievements of economic and social development in the 70th anniversary of the founding of New China*, which published on August 22, 2019 on the website of China’s Bureau of Statistics, “Steady growth in total population, significant improvement in population quality”—China’s population growth rate has remained steady at a growth rate of about 0.5% since 1991. However, the relative growth rate of the number of medical consultations is close to 5%, which is approximately 10 times the population growth rate. Therefore, the increase in the number of admissions has led to an increase in the workload of medical staff, which has led to a decline in the quality of medical services, mainly due to the mismatch between the supply of medical service resources and the people’s health demands in China. For follow-up studies on the quality of medical services that may be caused by the insufficient supply of medical resources, one should take into account the natural population growth rate, technological progress and the ratio of doctors and patients, and strive for an early solution to the problem of difficult and expensive access to medical care.

## Supporting information

S1 Table Summary statistics of diseases codes (ICD-10). (DOCX)

S1 File Data sample. (DTA)

S2 File Validity of the instrument variable. (DOCX)

S3 File Robustness. (DOCX)
