# Supplementary material for: Trading quality for quantity? Evidence from patient level data in China
Source: PLoS One. 2021 Sep 16;16(9):e0257127. doi: 10.1371/journal.pone.0257127 (PMC8445449; doi:10.1371/journal.pone.0257127)
Supplement: S1 Table — (DOCX) [file pone.0257127.s001.docx]

**S1 Table. Summary Statistics of Diseases Codes (ICD-10)**

| **Diseases codes** | **Frequency** | **Percentage** |
| --- | --- | --- |
| 9B | 126 | 0.01 |
| A0 | 7,326 | 0.85 |
| A1 | 10,646 | 1.23 |
| A3 | 412 | 0.05 |
| A4 | 1,821 | 0.21 |
| A6 | 191 | 0.02 |
| A8 | 928 | 0.11 |
| A9 | 144 | 0.02 |
| B0 | 13,346 | 1.54 |
| B1 | 2,842 | 0.33 |
| B2 | 1,447 | 0.17 |
| B3 | 132 | 0.02 |
| B4 | 322 | 0.04 |
| B9 | 676 | 0.08 |
| C0 | 107 | 0.01 |
| C1 | 8,457 | 0.98 |
| C2 | 8,724 | 1.01 |
| C3 | 283 | 0.03 |
| C4 | 212 | 0.02 |
| C5 | 6,693 | 0.78 |
| C6 | 2,188 | 0.25 |
| C7 | 3,391 | 0.39 |
| C8 | 2,788 | 0.32 |
| C9 | 4,328 | 0.5 |
| D0 | 404 | 0.05 |
| D1 | 3,130 | 0.36 |
| D2 | 6,033 | 0.7 |
| D3 | 565 | 0.07 |
| D4 | 520 | 0.06 |
| D5 | 1,782 | 0.21 |
| D6 | 6,386 | 0.74 |
| D7 | 126 | 0.01 |
| D8 | 108 | 0.01 |
| DB | 308 | 0.04 |
| E0 | 3,008 | 0.35 |
| E1 | 26,705 | 3.1 |
| E2 | 168 | 0.02 |
| E3 | 147 | 0.02 |
| E7 | 336 | 0.04 |
| E8 | 838 | 0.1 |
| F0 | 1,531 | 0.18 |
| F1 | 1,036 | 0.12 |
| F2 | 9,791 | 1.13 |
| F3 | 2,006 | 0.23 |
| F4 | 1,907 | 0.22 |
| F5 | 323 | 0.04 |
| F7 | 169 | 0.02 |
| F9 | 275 | 0.03 |
| G0 | 1,023 | 0.11 |
| G1 | 132 | 0.02 |
| G2 | 1,414 | 0.16 |
| G3 | 681 | 0.08 |
| G4 | 31,143 | 3.61 |
| G5 | 3,610 | 0.42 |
| G6 | 427 | 0.05 |
| G7 | 483 | 0.06 |
| G8 | 1,180 | 0.14 |
| G9 | 1,045 | 0.12 |
| H0 | 3,827 | 0.45 |
| H1 | 5,347 | 0.62 |
| H2 | 21,956 | 2.56 |
| H3 | 1,793 | 0.21 |
| H4 | 2,262 | 0.26 |
| H5 | 115 | 0.01 |
| H6 | 3,775 | 0.44 |
| H7 | 316 | 0.04 |
| H8 | 4,820 | 0.56 |
| H9 | 2,415 | 0.28 |
| I0 | 196 | 0.02 |
| I1 | 150 | 0.02 |
| I6 | 39,620 | 4.59 |
| I7 | 1,263 | 0.15 |
| I8 | 14,102 | 1.63 |
| I9 | 159 | 0.02 |
| K0 | 1,925 | 0.23 |
| K1 | 1,637 | 0.19 |
| K2 | 32,489 | 3.76 |
| K3 | 16,046 | 1.86 |
| K4 | 11,370 | 1.32 |
| K5 | 19,921 | 2.31 |
| K6 | 12,760 | 1.48 |
| K7 | 9,410 | 1.09 |
| K8 | 29,349 | 3.4 |
| K9 | 6,395 | 0.74 |
| L0 | 4,733 | 0.55 |
| L2 | 712 | 0.08 |
| L3 | 868 | 0.1 |
| L4 | 268 | 0.03 |
| L5 | 1,303 | 0.15 |
| L7 | 1,725 | 0.2 |
| L8 | 292 | 0.03 |
| L9 | 727 | 0.08 |
| M0 | 2,909 | 0.33 |
| M1 | 8,850 | 1.03 |
| M2 | 1,514 | 0.18 |
| M3 | 2,078 | 0.24 |
| M4 | 17,305 | 2.01 |
| M5 | 27,782 | 3.22 |
| M6 | 3,346 | 0.39 |
| M7 | 4,024 | 0.47 |
| M8 | 5,560 | 0.64 |
| M9 | 104 | 0.01 |
| N0 | 2,058 | 0.24 |
| N1 | 19,035 | 2.21 |
| N2 | 17,065 | 1.98 |
| N3 | 5,672 | 0.66 |
| N4 | 12,228 | 1.42 |
| N5 | 151 | 0.02 |
| N6 | 2,372 | 0.27 |
| N7 | 12,383 | 1.44 |
| N8 | 11,976 | 1.39 |
| N9 | 5,120 | 0.59 |
| O0 | 19,602 | 2.27 |
| O1 | 1,064 | 0.12 |
| O2 | 8,348 | 0.97 |
| O3 | 12,382 | 1.44 |
| O4 | 14,396 | 1.67 |
| O6 | 8,317 | 0.96 |
| O7 | 4,494 | 0.52 |
| O8 | 33,769 | 3.91 |
| O9 | 4,346 | 0.5 |
| P0 | 1,477 | 0.17 |
| P2 | 9,889 | 1.15 |
| P3 | 596 | 0.07 |
| P5 | 10,791 | 1.25 |
| Q1 | 799 | 0.09 |
| Q2 | 1,416 | 0.16 |
| Q3 | 282 | 0.03 |
| Q5 | 965 | 0.11 |
| Q6 | 543 | 0.06 |
| Q7 | 158 | 0.02 |
| Q8 | 481 | 0.06 |
| R0 | 4,102 | 0.48 |
| R1 | 2,529 | 0.29 |
| R2 | 1,139 | 0.13 |
| R3 | 735 | 0.09 |
| R4 | 861 | 0.1 |
| R5 | 4,023 | 0.47 |
| R6 | 915 | 0.11 |
| R7 | 109 | 0.01 |
| R9 | 2,608 | 0.3 |
| S0 | 20,855 | 2.42 |
| S1 | 612 | 0.07 |
| S2 | 7,709 | 0.89 |
| S3 | 7,974 | 0.92 |
| S4 | 6,187 | 0.72 |
| S5 | 4,271 | 0.5 |
| S6 | 5,330 | 0.62 |
| S7 | 6,155 | 0.71 |
| S8 | 7,866 | 0.91 |
| S9 | 3,335 | 0.39 |
| T0 | 3,497 | 0.39 |
| T1 | 4,002 | 0.46 |
| T2 | 1,200 | 0.14 |
| T3 | 370 | 0.04 |
| T5 | 985 | 0.11 |
| T6 | 3,673 | 0.43 |
| T7 | 419 | 0.05 |
| T8 | 1,755 | 0.2 |
| T9 | 734 | 0.09 |
| Y0 | 124 | 0.01 |
| Z0 | 1,332 | 0.15 |
| Z3 | 2,773 | 0.32 |
| Z4 | 11,958 | 1.39 |
| Z5 | 3,861 | 0.45 |
| Z7 | 617 | 0.07 |
| Z9 | 2,144 | 0.25 |
| Total | 862,722 |  |
