# Supplementary material for: Trading quality for quantity? Evidence from patient level data in China
Source: PLoS One. 2021 Sep 16;16(9):e0257127. doi: 10.1371/journal.pone.0257127 (PMC8445449; doi:10.1371/journal.pone.0257127)
Supplement: S2 File — (DOCX) [file pone.0257127.s003.docx]

**Validity of The Instrument Variable**

We selected the air quality index(AQI) as the instrumental variable(IV) of the number of patients per day to meet the primary requirements for using an IV method in a linear model: First, the AQI index was an exogenous variable that did not have any direct effect on the explained variables and other explanatory variables in the empirical model; Second, the AQI index (IV in Fig 1) was presumed to influence the explained variables (Y in Fig 1) exclusively via its effect on the endogenous explanatory variable (X in Fig 1).


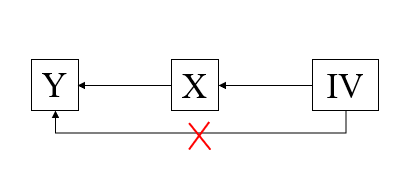


**Fig 1.** **Schematic Presentation Depicting the Instrumental Variable Assumption**

X= the number of patients admitted per day; Y= length of stay and hospital mortality rates, excluding the patients with stroke, heart disease, lung cancer and other diseases of the respiratory system; IV= daily air quality index (AQI).

**Table 1. First Stage for Number of Patients Admitted Per Day for IV Regressions of Log Length of Stay**

|  | **(1)** | **(2)** | **(3)** |
| --- | --- | --- | --- |
| **AQI** | 0.0026 (0.0646)*** | 0.0026 (0.0004)*** | 0.0021 (0.0002)*** |
| **Patient X** |  |  |  |
| Age |  | -0.1377 (0.0007)*** | 0.0005 (0.0005) |
| Gender (ref. female) |  | 0.3144 (0.0225)*** | 0.0440 (0.0140)*** |
| Hospitalization times |  | 0.0000 (0.0000)*** | -0.0000 (0.0000)*** |
| Hospital admission |  | 0.8159 (0.0125)*** | 0.0416 (0.0088)*** |
| **Payment (ref. URBMI)** |  |  |  |
| UEBMI |  | 0.0186 (0.0306) | 0.0216 (0.0190) |
| NRCMS |  | 0.1623 (0.0460)*** | 0.1546 (0.0267)*** |
| Others |  | -2.2710 (0.0237)*** | -0.1373 (0.0186)*** |
| **Employment status (ref. employed)** |  |  |  |
| Unemployment |  | -0.8306 (0.0455)*** | 0.1005 (0.0267)*** |
| Farmer |  | -0.4466 (0.0353)*** | 0.1490 (0.0217)*** |
| Retire |  | 0.8228 (0.0513)*** | 0.0494 (0.0305) |
| Self-employment |  | -0.2008 (0.0548)*** | -0.0613 (0.0326)* |
| Others |  | 0.5308 (0.0311)*** | 0.0910 (0.0192)*** |
| **Marriage status (ref. married)** |  |  |  |
| Single |  | 0.2443 (0.0437)*** | 0.2130 (0.0329)*** |
| Widowed |  | -1.4635 (0.0607)*** | -0.1387 (0.0424)*** |
| Divorced |  | -0.4700 (0.1783)*** | -0.5260 (0.1260)*** |
| Others |  | -4.0786 (0.0570)*** | 0.1867 (0.0480)*** |
| **HospitalX (ref. tertiary)** |  |  |  |
| Primary |  |  | 0.4910 (0.1590)*** |
| Secondary |  |  | 2.1939 (0.9916)** |
| Others |  |  | -0.3749 (0.3365) |
| **Year** | Yes | Yes | Yes |
| **Month** | Yes | Yes | Yes |
| **Holiday** | Yes | Yes | Yes |
| **ICD** | No | Yes | Yes |
| **Patient X** | No | Yes | Yes |
| **Hospital FE** | No | No | Yes |
| **F-test** | 48.91 | 54.62 | 91.46 |
| **R^2^** | 0.0289 | 0.0886 | 0.6509 |
| **Obs.** | 862,722 | 862,722 | 862,722 |
| *Notes:* Standard errors in parentheses.  Significance level: *** p <0.01, ** p <0.05, * p <0.1.  Patient’s personal control variables include age, gender, payment method, admission channel, occupation, marriage, and hospitalization times; Hospital control variables include hospital tier; The results of all the control variables are consistent with expectations; Due to space limitations, no results are reported in the table.  AQI: air quality index; Hospital FE: hospital fixed effects; | | | |

**Table 2. First Stage for Number of Patients Admitted Per Day for IV Regressions of Hospital Mortality**

|  | **(1)** | **(2)** | **(3)** |
| --- | --- | --- | --- |
| **AQI** | 0.0026 (0.0004)*** | 0.0026 (0.0004)*** | 0.0021 (0.0002)*** |
| **Patient X** |  |  |  |
| Age |  | -0.1377 (0.0007)*** | 0.0005 (0.0005) |
| Gender (ref. female) |  | 0.3130 (0.0225)*** | 0.0440 (0.0140)*** |
| Hospitalization times |  | 0.0000 (0.0000)*** | -0.0000 (0.0000)*** |
| Hospital admission |  | 0.8179 (0.0125)*** | 0.0418 (0.0088)*** |
| **Payment (ref. URBMI)** |  |  |  |
| UEBMI |  | 0.0242 (0.0306) | 0.0216 (0.0190) |
| NRCMS |  | 0.1652 (0.0460)*** | 0.1545 (0.0267)*** |
| Others |  | -2.3723 (0.0237)*** | -0.1367 (0.0186)*** |
| **Employment status (ref. employed)** |  |  |  |
| Unemployment |  | -0.8319 (0.0454)*** | 0.1004 (0.0267)*** |
| Farmer |  | -0.4444 (0.0353)*** | 0.1489 (0.0217)*** |
| Retire |  | 0.8118 (0.0513)*** | 0.0493 (0.0305) |
| Self-employment |  | -0.2008 (0.0512)*** | -0.0614 (0.0326)* |
| Others |  | 0.5314 (0.0311)*** | 0.0908 (0.0192)*** |
| **Marriage status (ref. married)** |  |  |  |
| Single |  | 0.2492 (0.0437)*** | 0.2129 (0.0329)*** |
| Widowed |  | -1.4618 (0.0607)*** | -0.1387 (0.0424)*** |
| Divorced |  | -0.4711 (0.1782)*** | -0.5256 (0.1260)*** |
| Others |  | -4.0781 (0.0570)*** | 0.1869 (0.0480)*** |
| **HospitalX (ref. tertiary)** |  |  |  |
| Primary |  |  | -0.0042 (0.1588)*** |
| Secondary |  |  | 2.1936 (0.9916)** |
| Others |  |  | -0.0500 (0.3641) |
| **Year** | Yes | Yes | Yes |
| **Month** | Yes | Yes | Yes |
| **Holiday** | Yes | Yes | Yes |
| **ICD** | No | Yes | Yes |
| **Patient X** | No | Yes | Yes |
| **Hospital FE** | No | No | Yes |
| **F-test** | 49.31 | 55.08 | 91.18 |
| **R^2^** | 0.0288 | 0.0881 | 0.6509 |
| **Obs.** | 862,722 | 862,722 | 862,722 |
| *Notes:* Standard errors in parentheses.  Significance level: *** p <0.01, ** p <0.05, * p <0.1.  Patient’s personal control variables include age, gender, payment method, admission channel, occupation, marriage, and hospitalization times; Hospital control variables include hospital tier; The results of all the control variables are consistent with expectations; Due to space limitations, no results are reported in the table.  AQI: air quality index; Hospital FE: hospital fixed effects; | | | |

**Table 3. First Stage for Number of Patients Admitted Per Day for IV Regressions of Log Length of Stay for Different Hospital Tiers**

|  | **(1)Primary hospitals** | **(2)Secondary hospitals** | **(3)Tertiary hospitals** | **(4)Others** |
| --- | --- | --- | --- | --- |
| **AQI** | -0.0012 (0.0004)** | 0.0007 (0.0002)*** | 0.0034 (0.0004)*** | 0.0028 (0.0004)*** |
| **Patient X** |  |  |  |  |
| Age | 0.0034 (0.0009)*** | 0.0003 (0.0004) | 0.0003 (0.0011) | 0.0002 (0.0006) |
| Gender (ref. female) | 0.0857 (0.0305)*** | -0.1453 (0.0273)*** | 0.1346 (0.0288)*** | 0.0476 (0.0180)*** |
| Hospitalization times | 0.0000 (0.0000)*** | 0.0000 (0.0000)*** | 0.0149 (0.0113) | -0.0000 (0.0000)*** |
| Hospital admission | -0.1484 (0.0497)*** | -0.0536 (0.0094)*** | 0.1669 (0.0170)*** | -0.1449 (0.0197)*** |
| **Payment (ref. URBMI)** |  |  |  |  |
| UEBMI | 0.0699 (0.0389)* | -0.0542 (0.0212)** | 0.0869 (0.0342) | -0.0553 (0.0203)*** |
| NRCMS | -0.1664 (0.0920)* | -0.1453 (0.0273)*** | -0.1224 (0.0475)* | -0.1124 (0.0203)*** |
| Others | 0.3331 (0.1118)*** | -0.0415 (0.0210)** | -0.2201 (0.0321)*** | -0.1830 (0.0266)*** |
| **Employment status (ref. employed)** |  |  |  |  |
| Unemployment | 0.2062 (0.2082) | -0.1170 (0.0302)*** | 0.2679 (0.0491)*** | -0.0079 (0.0446) |
| Farmer | 0.1308 (0.0474)*** | 0.1192 (0.0224)*** | 0.3036 (0.0441)*** | -0.1699 (0.0427)*** |
| Retire | 0.0096 (0.0703) | -0.1007 (0.0320)*** | 0.1441 (0.0559)* | 0.0622 (0.0412) |
| Self-employment | 0.0958 (0.0600) | -0.1831 (0.0375)*** | 0.1251 (0.0582)** | -0.1603 (0.0425)*** |
| Others | -0.0150 (0.0354) | 0.0521 (0.0198)*** | 0.2050 (0.0366)*** | -0.1735 (0.0361)*** |
| **Marriage status (ref. married)** |  |  |  |  |
| Single | 0.3330 (0.0869)*** | 0.0880 (0.0233)*** | 0.3574 (0.0867)*** | 0.0315 (0.0415) |
| Widowed | 0.0809 (0.1194) | -0.0611 (0.0351)* | -0.3181 (0.1197)*** | 0.0311 (0.0511) |
| Divorced | -0.1941 (0.2955) | -0.0781 (0.0655) | 1.4346 (0.3217)*** | 0.2993 (0.1513)** |
| Others | 0.0148 (0.0733) | -0.0056 (0.0296) | 0.4592 (0.1767)*** | 0.2926 (0.0442)*** |
| **F-test** | 4.96 | 11.43 | 57.09 | 63.31 |
| **R^2^** | 0.4617 | 0.5530 | 0.5473 | 0.4870 |
| **Obs.** | 22,457 | 385,876 | 369,150 | 85,239 |
| *Notes:* Standard errors in parentheses.  Significance level: *** p <0.01, ** p <0.05, * p <0.1.  Patient’s personal control variables include age, gender, payment method, admission channel, occupation, marriage, and hospitalization times; Hospital control variables include hospital tier; The results of all the control variables are consistent with expectations; Due to space limitations, no results are reported in the table.  AQI: air quality index. | | | | |

**Table 4. First Stage for Number of Patients Admitted Per Day for IV Regressions of Hospital Mortality for Different Hospital Tiers**

|  | **(1)Primary hospitals** | **(2)Secondary hospitals** | **(3)Tertiary hospitals** | **(4)Others** |
| --- | --- | --- | --- | --- |
| **AQI** | -0.0012 (0.0004)** | 0.0007 (0.0002)*** | 0.0034 (0.0004)*** | 0.0028 (0.0004)*** |
| **Patient X** |  |  |  |  |
| Age | 0.0034 (0.0009)*** | 0.0003 (0.0004) | 0.0003 (0.0011) | 0.0002 (0.0006) |
| Gender (ref. female) | 0.0857 (0.0305)*** | -0.0310 (0.0124)** | 0.1346 (0.0288)*** | 0.0476 (0.0180)*** |
| Hospitalization times | 0.0000 (0.0000)*** | 0.0000 (0.0000)*** | 0.0149 (0.0113) | -0.0000 (0.0000)*** |
| Hospital admission | -0.1484 (0.0497)*** | -0.0537 (0.0093)*** | 0.1675 (0.0170)*** | -0.1449 (0.0197)*** |
| **Payment (ref. URBMI)** |  |  |  |  |
| UEBMI | 0.0699 (0.0389)* | -0.0542 (0.0212)** | 0.0868 (0.0342)** | -0.0553 (0.0203)*** |
| NRCMS | -0.1664 (0.0920)* | -0.1452 (0.0273)*** | -0.1222 (0.0475)*** | -0.1124 (0.0230)*** |
| Others | 0.3331 (0.1118)*** | -0.0415 (0.0210)** | -0.2183 (0.0321)*** | -0.1830 (0.0266)*** |
| **Employment status (ref. employed)** |  |  |  |  |
| Unemployment | 0.2062 (0.2082) | -0.1170 (0.0302)*** | 0.2679 (0.0490)*** | -0.0079 (0.0446) |
| Farmer | 0.1308 (0.0474)*** | 0.1192 (0.0224)*** | 0.3028 (0.0441)*** | -0.1699 (0.0427)*** |
| Retire | 0.0096 (0.0703) | -0.1005 (0.0319)*** | 0.1438 (0.0559)*** | 0.0622 (0.0412) |
| Self-employment | 0.0958 (0.0600) | -0.1831 (0.0375)*** | 0.1247 (0.0582)** | -0.1603 (0.0425)*** |
| Others | -0.0150 (0.0354) | 0.0521 (0.0198)*** | 0.2043 (0.0366)*** | -0.1735 (0.0361)*** |
| **Marriage status (ref. married)** |  |  |  |  |
| Single | 0.3330 (0.0869)*** | 0.0881 (0.0233)*** | 0.3569 (0.0866)*** | 0.0315 (0.0415) |
| Widowed | 0.0809 (0.1194) | -0.0611 (0.0351)* | -0.3185 (0.1197)*** | 0.0311 (0.0511) |
| Divorced | -0.1941 (0.2955) | -0.0776 (0.0654) | 1.4331 (0.3214)*** | 0.2993 (0.1513)** |
| Others | 0.0148 (0.0733) | -0.0052 (0.0296) | 0.4590 (0.1767)*** | 0.2926 (0.0442)*** |
| **F-test** | 4.96 | 11.39 | 56.87 | 63.31 |
| **R^2^** | 0.4617 | 0.5531 | 0.5476 | 0.4870 |
| **Obs.** | 22,457 | 385,876 | 369,150 | 85,239 |
| *Notes:* Standard errors in parentheses.  Significance level: *** p <0.01, ** p <0.05, * p <0.1.  Patient’s personal control variables include age, gender, payment method, admission channel, occupation, marriage, and hospitalization times; Hospital control variables include hospital tier; The results of all the control variables are consistent with expectations; Due to space limitations, no results are reported in the table.  AQI: air quality index. | | | | |
