# Supplementary material for: Trading quality for quantity? Evidence from patient level data in China
Source: PLoS One. 2021 Sep 16;16(9):e0257127. doi: 10.1371/journal.pone.0257127 (PMC8445449; doi:10.1371/journal.pone.0257127)
Supplement: S3 File — (DOCX) [file pone.0257127.s004.docx]

# Robustness

| **Table1. Descriptive Statistics of Daily Weather Conditions from January 2014 to June 2019** | | | | | | | |
| --- | --- | --- | --- | --- | --- | --- | --- |
|  | **Mean** | **SD** | **Min** | **P25** | **P50** | **P75** | **Max** |
| **Temperature (℃)** | 19.22 | 7.70 | 1.20 | 12.20 | 19.30 | 25.00 | 36.50 |
| **Humidity (%)** | 75.65 | 11.24 | 37.00 | 68.00 | 77.00 | 84.00 | 97.00 |
| **Wind (m/s)** | 1.34 | 0.39 | 0.30 | 1.1 | 1.3 | 1.5 | 3.7 |
| **Pressure (hpa)** | 983.18 | 8.50 | 962.60 | 976.00 | 983.00 | 989.80 | 1012.3 |
| **Precipitation (mm)** | 3.82 | 9.63 | 0 | 0 | 0 | 2.8 | 111.2 |
| **Sunshine hours** | 2.93 | 3.95 | 0 | 0 | 0 | 6.2 | 12.8 |

| **Table2. Robustness Checks for IV Regression of Log Length of Stay** | | | |
| --- | --- | --- | --- |
|  | **(1)** | **(2)** | **(3)** |
| Panel A: Two-Stage Least Squares | | | |
| **N** | -0.0147 (0.0075)** | -0.0446 (0.0091)*** | -0.0588 (0.0109)*** |
| **Patient X** |  |  |  |
| Age |  | 0.0030 (0.0002)*** | 0.0037 (0.0001)*** |
| Gender(ref. female) |  | 0.0092 (0.0020)*** | 0.0135 (0.0020)*** |
| Hospital admission |  | 0.0221 (0.0061)*** | 0.0072 (0.0014)*** |
| **Payment (ref. URBMI)** |  |  |  |
| UEBMI |  | 0.0645 (0.0025)*** | 0.0619 (0.0025)*** |
| NRCMS |  | -0.0158 (0.0039)*** | -0.0226 (0.0037)*** |
| Others |  | -0.1028 (0.0201)*** | -0.0005 (0.0030)*** |
| **Employment status (ref. employed)** |  |  |  |
| Unemployment |  | -0.0665 (0.0077)*** | -0.0145 (0.0037)*** |
| Farmer |  | -0.0758 (0.0066)*** | -0.0548 (0.0034)*** |
| Retire |  | 0.0900 (0.0077)*** | 0.0769 (0.0042)*** |
| Self-employment |  | -0.0155 (0.0053)*** | -0.0258 (0.0047)*** |
| Others |  | -0.0050 (0.0040)*** | -0.0305 (0.0029)*** |
| **Marriage status (ref. married)** |  |  |  |
| Single |  | 0.0155 (0.0036)*** | 0.0187 (0.0041)*** |
| Widowed |  | -0.061 (0.0132)*** | -0.0116 (0.0059)* |
| Divorced |  | 0.0407 (0.0120)*** | 0.0850 (0.0123)*** |
| Others |  | -0.1437 (0.0326)*** | -0.0141 (0.0055)** |
| **HospitalX (ref. tertiary)** |  |  |  |
| Primary |  |  | -0.9774 (0.1302)*** |
| Secondary |  |  | 0.1967 (0.2228) |
| Others |  |  | 0.2713 (0.1453)* |
| **Weather conditions** |  |  |  |
| Temperature | -0.0007 (0.0006) | -0.0011 (0.0006)** | -0.0008 (0.0005) |
| Humidity | -0.0002 (0.0001) | -0.0001 (0.0001) | 0.0000 (0.0001) |
| Wind | -0.0001 (0.0001) | 0.0000 (0.0000) | 0.0000 (0.0000) |
| Pressure | 0.0011(0.0003)*** | 0.0006 (0.0003)*** | 0.0004 (0.0003) |
| Precipitation | -0.0001 (0.0001) | -0.0003 (0.0001)*** | -0.0006 (0.0001)*** |
| Sunshine hours | 0.0003 (0.0004) | 0.0001 (0.0003) | 0.0003 (0.0003) |
| **Hospitalization times** | No | Yes | Yes |
| **ICD10** | No | Yes | Yes |
| **Hospital_FE** | No | No | Yes |
| **Year** | Yes | Yes | Yes |
| **Month** | Yes | Yes | Yes |
| **Holiday** | Yes | Yes | Yes |
| **MSE** | 0.58 | 0.52 | 0.47 |
| **F-statistic** | 46.56 | 228.72 | 334.05 |
| Panel B: First Stage for Number of Patients Admitted Per Day | | | |
| **AQI** | 0.0043 (0.0004)*** | 0.0034 (0.0004)*** | 0.0027 (0.0003)*** |
| **F-statistic** | 97.10 | 79.44 | 111.06 |
| **R**^2^ | 0.0284 | 0.2046 | 0.6536 |
| **Obs.** | 862,722 | 862,722 | 862,722 |
| *Notes*: Standard errors in parentheses.  Significance level: *** p <0.01, ** p <0.05, * p <0.1.  Patient’s personal control variables include age, gender, payment method, admission channel, occupation, marriage, and hospitalization times; Hospital control variables include hospital tier; The results of all the control variables are consistent with expectations; Due to space limitations, no results are reported in the table.  N: number of patients admitted per day; NRCMS: the new rural cooperative medical insurance; UEBMI: the basic medical insurance for urban employees; URBMI: the basic medical insurance for urban residents; Hospital_FE: hospital fixed effects; MSE: mean square error; AQI: daily air quality index; Obs.: the number of observations. | | | |

| **Table3. Robustness Checks for IV Regression of Hospital Mortality** | | | |
| --- | --- | --- | --- |
|  | **(1)** | **(2)** | **(3)** |
| Panel A: Two-Stage Least Squares | | | |
| **N** | 0.0098 (0.0024)*** | 0.0118 (0.0029)*** | 0.0161 (0.0030)*** |
| **Patient X** |  |  |  |
| Age |  | 0.0002 (0.0000)*** | -0.0000 (0.0000)* |
| Gender(ref. female) |  | 0.0030 (0.0006)*** | 0.0003 (0.0005) |
| Hospital admission |  | -0.0638 (0.0020)*** | -0.0559 (0.0005)*** |
| **Payment (ref. URBMI)** |  |  |  |
| UEBMI |  | -0.0056 (0.0009)*** | 0.0009 (0.0007) |
| NRCMS |  | -0.0096 (0.0012)*** | 0.0027 (0.0009)*** |
| Others |  | -0.0051 (0.0065)*** | 0.0108 (0.0008)*** |
| **Employment status (ref. employed)** |  |  |  |
| Unemployment |  | 0.0193 (0.0025)*** | 0.0179 (0.0010)*** |
| Farmer |  | 0.0170 (0.0021)*** | 0.0092 (0.0009)*** |
| Retire |  | 0.0239 (0.0025)*** | 0.0330 (0.0012)*** |
| Self-employment |  | -0.0029 (0.0015)* | 0.0041 (0.0012)*** |
| Others |  | 0.0052 (0.0012)*** | 0.0140 (0.0007)*** |
| **Marriage status (ref. married)** |  |  |  |
| Single |  | -0.0023 (0.0011)** | -0.0046 (0.0011)*** |
| Widowed |  | 0.0139 (0.0042)*** | 0.0016 (0.0015) |
| Divorced |  | 0.0301 (0.0035)*** | 0.0140 (0.0033)*** |
| Others |  | 0.0834 (0.0106)*** | 0.0122 (0.0016)*** |
| **HospitalX (ref. tertiary)** |  |  |  |
| Primary |  |  | -0.0650 (0.0064)*** |
| Secondary |  |  | -0.1368 (0.0175)*** |
| Others |  |  | -0.0480 (0.0070)*** |
| **Weather conditions** |  |  |  |
| Temperature | 0.0002 (0.0002) | 0.0001 (0.0002) | 0.0000 (0.0001) |
| Humidity | 0.0000 (0.0000) | -0.0000 (0.0000) | -0.0001 (0.0000)* |
| Wind | 0.0000 (0.0000) | 0.0000 (0.0000) | 0.0000 (0.0000) |
| Pressure | -0.0004 (0.0001)*** | -0.0003 (0.0001)*** | -0.0003 (0.0001)*** |
| Precipitation | 0.0000 (0.0000) | 0.0001 (0.0000) | 0.0001 (0.0000)*** |
| Sunshine hours | 0.0001 (0.0001) | 0.0001 (0.0001) | -0.0000 (0.0001) |
| **Hospitalization times** | No | Yes | Yes |
| **ICD10** | No | Yes | Yes |
| **Hospital_FE** | No | No | Yes |
| **Year** | Yes | Yes | Yes |
| **Month** | Yes | Yes | Yes |
| **Holiday** | Yes | Yes | Yes |
| **MSE** | 0.58 | 0.52 | 0.47 |
| **F-statistic** | 52.27 | 23.43 | 83.87 |
| Panel B: First Stage for Number of Patients Admitted Per Day | | | |
| **AQI** | 0.0043 (0.0004)*** | 0.0033 (0.0004)*** | 0.0027 (0.0003)*** |
| **F-statistic** | 97.47 | 80.98 | 109.78 |
| **R**^2^ | 0.03 | 0.20 | 0.65 |
| **Obs.** | 862,722 | 862,722 | 862,722 |
| *Notes*: Standard errors in parentheses.  Significance level: *** p <0.01, ** p <0.05, * p <0.1.  Patient’s personal control variables include age, gender, payment method, admission channel, occupation, marriage, and hospitalization times; Hospital control variables include hospital tier; The results of all the control variables are consistent with expectations; Due to space limitations, no results are reported in the table.  N: number of patients admitted per day; NRCMS: the new rural cooperative medical insurance; UEBMI: the basic medical insurance for urban employees; URBMI: the basic medical insurance for urban residents; Hospital_FE: hospital fixed effects; MSE: mean square error; AQI: daily air quality index; Obs.: the number of observations. | | | |

| **Table4. Robustness Checks for IV Regression of Log Length of Stay for Different Hospital Tiers** | | | | |
| --- | --- | --- | --- | --- |
|  | **(1) Primary hospitals** | **(2) Secondary hospitals** | **(3) Tertiary hospitals** | **(4) Others** |
| Panel A: Two-Stage Least Squares | | | | |
| **N** | 0.465 (1.0711) | -0.154 (0.0495)*** | -0.0450 (0.0106)*** | 0.0310 (0.0316) |
| **Weather conditions** |  |  |  |  |
| Temperature | -0.001 (0.0046) | -0.005 (0.0018)*** | 0.0001 (0.0008) | 0.0005 (0.0015) |
| Humidity | 0.001 (0.0016) | -0.000 (0.0002) | 0.0002 (0.0002) | 0.0004 (0.0003) |
| Wind | 0.008 (0.0170) | 0.000 (0.0000) | 0.0001 (0.0001) | -0.0005 (0.0007) |
| Pressure | -0.015 (0.0341) | -0.005 (0.0015)*** | 0.0023 (0.0006)*** | 0.0000 (0.0007) |
| Precipitation | 0.002 (0.0053) | -0.001 (0.0005)*** | -0.0007 (0.0002)*** | 0.0002 (0.0003) |
| Sunshine hours | 0.007 (0.0124) | -0.001 (0.0007) | 0.0013 (0.0006)** | -0.0001 (0.0010) |
| **MSE** | 59.76 | 4.24 | 66.30 | 1.10 |
| **F** | 5.40 | 46.57 | 62.03 | 86.87 |
| Panel B: First Stage for Number of Patients Admitted Per Day | | | | |
| **AQI** | -0.0003 (0.0005) | 0.001 (0.0002)*** | 0.0043 (0.0005)*** | 0.0025 (0.0004)*** |
| **F-statistic** | 0.26 | 21.55 | 68.41 | 36.59 |
| **R**^2^ | 0.4892 | 0.5689 | 0.5563 | 0.5130 |
| **Obs.** | 22,457 | 385,876 | 369,150 | 85,239 |
| *Notes*: Standard errors in parentheses.  Significance level: *** p <0.01, ** p <0.05, * p <0.1.  Patient’s personal control variables include age, gender, payment method, admission channel, occupation, marriage, and hospitalization times; Hospital control variables include hospital tier; The results of all the control variables are consistent with expectations; Due to space limitations, no results are reported in the table.  N: number of patients admitted per day; MSE: mean square error; AQI: daily air quality index; Obs.: the number of observations. | | | | |

| **Table5. Robustness Checks for IV Regression of Hospital Mortality for Different Hospital Tiers** | | | |  |
| --- | --- | --- | --- | --- |
| **Variable** | **(1) Primary hospitals** | **(2) Secondary hospitals** | **(3) Tertiary hospitals** | **(4) Others** |
| Panel A: Two-Stage Least Squares | | | | |
| **N** | 0.1002 (0.2681) | 0.5489 (0.0164)*** | 0.0095 (0.0024)*** | -0.0080 (0.0107) |
| **Weather conditions** |  |  |  |  |
| temperature | -0.0005 (0.0012) | 0.0013 (0.0006)** | 0.0002 (0.0002) | 0.0001 (0.0001) |
| humidity | -0.0000 (0.0004) | 0.0001 (0.0001) | -0.0002 (0.0000)*** | 0.0001 (0.0001) |
| wind | 0.0013 (0.0043) | 0.0000 (0.0000) | 0.0000 (0.0000) | -0.0001 (0.0002) |
| pressure | -0.0035 (0.0086) | 0.0015 (0.0005)*** | -0.0005 (0.0001)*** | -0.0003 (0.0002) |
| precipitation | 0.0005 (0.0013) | 0.0005 (0.0002)*** | 0.0001 (0.0000)*** | -0.0002 (0.0001)*** |
| sunshine hours | 0.0011 (0.0031) | 0.0007 (0.0002)*** | -0.0004 (0.0001)*** | -0.0001 (0.0004) |
| **MSE** | 2.49 | 0.48 | 93.51 | 0.08 |
| **F** | 79.25 | 26.58 | 67.61 | 107.362 |
| Panel B: First Stage for Number of Patients Admitted Per Day | | | | |
| **AQI** | -0.0003 (0.0005) | 0.0010 (0.0002)*** | 0.0043 (0.0005)*** | 0.0025 (0.0004)*** |
| **F-statistic** | 0.26 | 21.50 | 68.31 | 36.59 |
| **R**^2^ | 0.49 | 0.57 | 0.56 | 0.51 |
| **Obs.** | 22,457 | 385,876 | 369,150 | 85,239 |
| *Notes*: Standard errors in parentheses.  Significance level: *** p <0.01, ** p <0.05, * p <0.1.  Patient’s personal control variables include age, gender, payment method, admission channel, occupation, marriage, and hospitalization times; Hospital control variables include hospital tier; The results of all the control variables are consistent with expectations; Due to space limitations, no results are reported in the table.  N: number of patients admitted per day; MSE: mean square error; AQI: daily air quality index; Obs.: the number of observations. | | | | |
